# Short-term and mid-term effects of thoracoscopic repair of esophageal atresia: No anastomotic leaks or conversions to open technique

**DOI:** 10.3389/fsurg.2022.1009448

**Published:** 2022-11-23

**Authors:** Przemyslaw Galazka, Dominika Skinder, Jan Styczynski

**Affiliations:** ^1^ Department of General and Oncologic Surgery for Children and Adolescents, Nicolaus Copernicus University Torun, Collegium Medicum, Bydgoszcz, Poland; ^2^ Department of Pediatric Hematology and Oncology, Nicolaus Copernicus University Torun, Collegium Medicum, Bydgoszcz, Poland

**Keywords:** esophageal atresia, thoracoscopic repair, newborn surgery, tracheoesophageal fistula, esophageal anastomosis, risk factors, overall survival

## Abstract

The frequency rate of esophageal anastomosis leaks after thoracoscopic correction of esophageal atresia (EA) in the current literature is reported as 5.6%–24.7% and a conversion rate of 2%–53%. The objective of this retrospective study was to examine the characteristics of EA and analysis of the safety and efficacy of EA repair with the use of the thoracoscopic approach in a single academic center, as well as risk factors analysis in the context of short-term and mid-term follow-up status. A retrospective analysis of the management of all consecutive newborns affected by EA hospitalized in our department over a period between 2013 and 2022, including preoperative, perioperative, and postoperative management, together with the outcome, complications and long-term follow-up status was performed. A total of 38 patients with a median birth weight of 2,570 g (range; 1,020–3,880) were treated over the study period, including 30/38 (78.9%) with additional congenital anomalies. Overall, 30 patients underwent primary anastomosis of the esophagus and eight underwent a multistaged procedure, with or without an initial ligation of the tracheoesophageal fistula and delayed primary anastomosis. Overall survival for all patients was 0.894 ± 0.050, with a median follow-up of 4.5 years. We noted neither anastomotic leaks nor conversions to open technique in our cohort. Implementation of vancomycin prophylaxis was successful in preventing postoperative central venous access-related infectious complications. At the end of the follow-up, 85% of patients have a Lansky performance score ≥80. Risk factors analysis for length of hospitalization, overall survival, Lansky performance status, and neurological impairment were analyzed. In conclusion, we have found that the outcome of thoracoscopic repair of EA in terms of surgery-dependent morbidity (anastomosis leakage, conversion rate to open surgery), provides benefit to those previously reported in the literature, regardless of the prognostic criteria of the classification system.

## Introduction

Primary repair of esophageal atresia (EA) surgery includes division of the tracheoesophageal fistula (TEF) and precise esophageal anastomosis. Recent studies have shown that survival rates exceeded over 90% (1–3). In many pediatric surgery centers, in the case of a newborn with EA TEF, still, the mainstay of treatment is thoracotomy with an extrapleural ligation of the fistula and end-to-end anastomosis of esophageal segments. Although thoracoscopic correction of EA TEF has been performed since 1999 (4, 5), this method is not been widely adopted and high conversion rates are still reported (5).

According to recent publications, morbidity after primary repair of proximal EA and distal TEF patients is still substantial and many common practices do not appear to reduce complications (Table 1) (6). Therefore, attempts need to be made to improve outcomes after EA treatment in terms of surgical complications (3). Classification systems based on preoperative factors are being used for patients with EA predicting mortality. These classifications not only predict survival based on these factors but also indicate in some centers the choice of surgical approach (3, 7, 8).

**Table 1:** Reported outcomes of surgical management of esophageal atresia in recent studies.

Source	Number of patients	Recurrent anastomosis stricture	Fundoplication rate	Gastrostomy	Anastomotic leak	Recurrent fistula	Mortality	Thoracoscopic method	Conversion rate
Etchill et al. 2021 (5)	855	—	—	—	—	—	15 (1.8%)	133 (15.6%)	70 (53%)
Lal et al. 2018 (6)	181	127 (43%)	—	—	54 (18%)	15 (5%)	17 (6%)	27 (9%)	—
Son et al. 2021 (19)	41	16 (39%)	10 (24%)	—	5 (12%)	—	1 (2%)	41 (100%)	1 (2%)
Elhattab et al 2020 (20)	47	33.3%	—	—	8.9%	—	—	—	—
Way et al. 2019 (21)	Meta-analysis	202/660 (30.6%)	(79/341) 23.1%	—	(91/728) 12.5%	11/414 (2.7%)	24/761 (3.2%)	100%	83/833 (9.9%)
Bence et al. 2021 (22)	170	30%	—	—	24.7%	—	—	26 (15.3%)	—
Sfeir et al. 2021 (23)	1,008 (various data)	183/917 (19.9%)	8.6%	13%	20/359 (5.6%)	28/868 (3.2%)	48/948 (5%)	0%	—

Based on the concept of reported use of minimally invasive surgery in endoscopic correction of esophageal atresia (9–11), a complex standardized management strategy has been introduced in newborns with esophageal atresia in many centers. Standardization was adopted for the treatment of all newborns diagnosed with EA and the surgical treatment was based on a thoracoscopic approach, together with standardization of perioperative management (12–14).

The primary objective of this retrospective study was to the characteristics of EA and analysis of the safety and efficacy of EA repair with the use of thoracoscopy approach in a single academic center. The secondary objective was the risk factors analysis in the context of short-term and mid-term follow-up status. With our experience in minimally invasive surgery (15–18), we present our data showing that outcome in terms of surgery-dependent morbidity of patients with EA treated with thoracoscopic method might improve previously reported results (Table 1) (5, 6, 19–23) in the context of a lower rate of leakage and other complications, such as conversion to open surgery, regardless of the prognostic criteria of the classification system.

## Materials and methods

### Study design

We retrospectively analyzed clinical data of all consecutive newborns affected by EA hospitalized in our department over a period between 2013 and 2022. Preoperative, perioperative, and postoperative management together with outcome, complications, and long-term follow-up status were assessed. All consecutive patients with EA were included in the study and had surgical repair of their congenital defect defined as the esophageal reconstruction with or without ligation of TEF within the first 2 months of life. None of the operated children was excluded from the analysis. Patient demographics, operative data, complications, and associated anomalies were noted. Children with EA were scored under each of the three classification systems (Spitz, Montreal, and Waterston) (24–27). The inclusion of patients was closed on March 31, and follow-up was on June 30, 2022.

### Preoperative clinical management

Whenever necessary, infants were mechanically ventilated and administered vasopressors prior or during surgery. In children <2,500 g, with prematurity or congenital heart defect, preoperative mechanical ventilation was always considered.

After stabilization of cardiopulmonary status, preoperative screening for coexisting congenital anomalies was conducted together with chest x-ray with safe injection of 1–2 ml of water-soluble contrast in order to improve visualization. The timing of surgery was scheduled according to cardiopulmonary stabilization and/or surgical urgency. Regardless of suspicion of type C or other forms of EA, an endoscopic correction was used as the preferred approach. Patients underwent diagnostic bronchoscopy prior or after surgical treatment, when necessary. Preoperative indications for bronchoscopy were present in case of any doubts on the pouchogram study (e.g., IVth type of laryngoesophageal cleft, H-type fistula). Postoperative indications included signs and symptoms of clinically significant tracheomalacia.

This procedure enabled the exclusion of multiple fistulae or laryngotracheal clefts. Classification of subtypes of EA was based on the classification of Gross (28–30). Additional surgical procedures: simultaneous or in the additional surgical procedure, because of additional congenital malformation, were conducted depending on the standard of care and surgical treatment practice.

### Operative procedures

All newborns with EA TEF have a central venous catheter (CVC) inserted percutaneously. Patients under general anesthesia were lying in prone position with a small towel placed under the right half of the body. In all cases, three ports were used: the first 5 mm port was inserted anteriorly to the tip of the scapula (and was used for clips application, for other manipulations a 3 mm overlay was used), the second 5 mm port was placed two intercostal spaces below in the middle axillary line and was used for camera, and the third 3 mm placed laterally to the paravertebral line. The insufflation CO_2_ pressure was 4–6 mmHg, with minimal gas flow (1 L/min).

Anesthesiologic ventilation technique details included permission for primary adaptation for pneumothorax with complete reduction of the intrathoracic pressure as necessary, permissive hypercapnia or short-time desaturation up to 80% SatO_2_, which were reversible after pneumothorax reduction or gentle increase of ventilation rate without the raise of insufflation pressure.

TEF was ligated with one 5 mm Hem-o-lok. Care was taken to prepare both ends of the esophagus with minimal mobilization and handling (without unnecessary grasping or pulling). The anastomosis was created with 5-0 absorbable braided sutures with the use of the intracorporeal knot-tying technique and the use of the “sliding knot” technique of both esophageal ends approximation. In each case, a 6 Fr transanastomotic feeding tube was used, as suggested for thoracoscopic approach (14). Pleural cavity drainage was not used only in selected cases with a good general condition of anastomosis and overall good general condition of patients.

In case of difficult local circumstances, like very high (e.g., Th1 level) proximal esophagus, lack of stability during the procedure [very low birth weight (VLBW) infants, patients with cardiac anomalies with impact on hemodynamic stability], the multistage surgery was conducted. In the first stage, local preparation of both esophageal ends was conducted, if the present fistula was closed, and internal traction sutures were placed. Usually, two vascular 4-0 monofilament stitches for traction were used.

### Postoperative management

Postoperative management was standardized but could be individualized for each patient after a multidisciplinary team meeting (surgeon, neonatologist, and intensivist). Postoperative management after EA thoracoscopic surgery included: in cases without significant tension anastomosis standard 24 h of paralysis and then slow withdrawal (usually after 2–3 days) from deep sedation. In case of significant tension on the anastomosis paralysis, it took 48–72 h with gradual withdrawal of the sedation. Patients were treated in a standard postoperative manner that enabled some newborns after cessation of paralysis gradually start to move and be restless, and to decrease the risk of unnecessary reintubation. After 2 days, thoracic drainage was removed. The pain was measured by nurses and intensivists from the intensive care unit (ICU) using Neonatal-Infant Pain Scale (NIPS). General management included supportive treatment, such as total parenteral nutrition, administration of intravenous antibiotics, a continuation of mechanical ventilation, pleural drainage. and sedation, whenever necessary. Anastomosis calibration was performed in each infant at 4 weeks after operation. If more than one dilatation was necessary, stricture was suspected.

### Antibacterial prophylaxis

Initially, standard antibiotic prophylaxis with amoxycillin and gentamicin was applied. In 2017, after a series of CVC sepsis during ICU stay and analysis of microbiologic situation, we administered widened antibiotic prophylaxis as a standard of care. Vancomycin at the dose 10 mg/kg i.v. was introduced at the time of CVC placement and was immediately withdrawn after CVC removal (usually a few days after postoperative x-ray esophagogram) (31, 32). In case of clinical signs suggesting a septic event, after collection of bacteriological cultures, empiric treatment with imipenem + cilastatin and fluconazole were administered. The correction was implemented as necessary after microbiologic test results. Each patient received GERD (gastroesophageal reflux disease) prophylactic treatment with a proton pump inhibitor i.v. sequentially changed for the oral route.

### Follow-up assessment

Overall condition was assessed during follow-up as an age-dependent pediatric development, overall neurological development, and ability to self-dependence. Special attention was paid to potential symptoms of dysphagia. Neurological development was performed by pediatric neurologists. Additionally, Lansky performance score (LPS) was assessed. Although LPS was originally designed and validated as a parent-reported measure to assess the performance status of their children with cancer, for the purpose of this study, we adopted the LPS and presented ranges from 10 to 100 to assess the developmental status of children after surgical treatment of EA, as previously it was used for other groups of patients (33, 34).

### Data analysis

We analyzed pre-, peri- and postoperative demographic, clinical, operative, laboratory, and outcome data in order to detect possible associations between variables. In perioperative data collection, we included demographics (gestational age, birth weight, associated congenital anomalies), clinical status (before and) at the time of the surgical procedure, as well as operative approach. In the postoperative period, we analyzed the occurrence of anastomotic leakage and stricture, other morbidities (pneumonia, length of ventilator dependence, length of hospital stay) and mortality, and the need for additional interventions during short-term and long-term follow-up.

### Primary and secondary endpoints of the study

The primary endpoint of the study was overall survival (OS). Secondary endpoints during the neonatal and infant period were the absence of anastomosis leakage and length of hospitalization. Secondary endpoints during follow-up were physical development, neurological complications, and Lansky performance score.

### Definitions

• Leakage was defined as the detection of contrast outside the esophageal lumen during control esophagogram that was routinely planned to be performed 10 days after surgery, unless reasonably delayed.
• Anastomosis stricture was defined as a narrowing at the level of the esophageal anastomosis.
• Fundoplication was performed if signs of “high” gastroesophageal reflux were present on the x-ray study and reflux symptoms were evident without improvement after medical treatment.
• Cardiac anomalies requiring medical or surgical treatment for heart failure were classified as major cardiac anomalies. Congenital heart disease was defined as clinically identified cardiac defects other than hemodynamically nonsignificant patent ductus arteriosus (PDA) and patent foramen ovale.

### Ethical considerations

All investigations and treatments were established clinical practices and were carried out according to accepted clinical practice and in compliance with the medical principles of the Declaration of Helsinki. Informed consent was obtained from all parents prior to treatment. The University Bioethical Committee approved the use of patient data for this study (Ref. KB 438/2022).

### Statistical analysis

Categorical variables were presented as numbers and percentages, while continuous variables were by medians and quartile values. Correlations between factors were analyzed with the Spearman rho coefficient. Cumulative incidence of final EA anastomosis, time of hospitalization, mean survival, and overall survival, were determined with the Kaplan–Meier method and compared by log-rank test. Univariate and multivariate logistic regression analyses were used to identify preoperative variables as predictors of study endpoints, with determination of odds ratio (OR) and 95% confidence interval (95%CI). Lists of potential risk factors analyzed are provided in respective sections. Statistical significance was defined for two-sided *p* < 0.05. SPSS 28 (IBM, Armonk, NY, United States) statistical package was used.

## Results

### Demographics

Overall, 38 patients were treated over the study period, including 27 males and 11 females (Table 2). The median gestational age was 37 (range, 28–41) weeks (Hbd, hebdomas) and median birth weight (b.w.) was 2,570 g (range 1,020–3,880 g). Overall, 15 (39.5%) patients were born prematurely with gestation age of less than 37 weeks. Three patients presented with b.w. < 1,500 g and were classified as VLBW.

**Table 2:** Demographic and clinical data of patients included in the study.

Characteristics	Value
Sex (*n*)	Female	11 (28.9%)
	Male	27 (71.1%)
Birth (*n*)	Full-term	23 (60.5%)
	Prematurity (33–36 Hbd)	12 (31.6%)
	Extreme prematurity (<32 Hbd)	3 (7.9%)
Gestational age (weeks)	Median (quartiles)	37 (34–40)
Birth weight (g)	Median (quartiles)	2,570 (1,960–3,200)
	<1,500	4
	1,500–2,000	6
	2,000–2,800	12
	>2,800	16
Birth length (cm)	Median (quartiles)	51 (48–55)
Cesarian section delivery (*n*)	Yes	24/38 (63.2%)
APGAR score	Median (quartiles)	8 (7–9)
Upper level of EA (Th vertebrae in x-ray)	Median (quartiles)	3 (2–3)
Disproportion of esophageal endings (*n* = 22)	Median (quartiles)	1.5:1 (1.5:1–2:1)
Coexisting congenital defects (*n*)	No	8 (21.1%)
	Yes	30 (78.9%)
Down syndrome	Yes	3 (7.9%)
Respiratory insufficiency before surgery	Yes	14 (36.8%)

### Coexisting congenital defects

In total, 30/38 (78.9%) of the patients had other congenital anomalies (Table 3). In 14 (36.8%) of patients cardiovascular congenital anomalies were present, in some of them multiple defects, including patent ductus arteriosus (PDA; *n* = 6; i.e., 15.8%), atrial septal defect (ASD; *n* = 10; i.e., 26.3%), ventricular septal defect (VSD; *n* = 6; i.e., 15.8%), ventriculoatrial septal defect (*n* = 1; i.e., 2.6%), pulmonary artery stenosis (*n* = 3; i.e., 7.9%), coarctation of aorta (CoA; *n* = 2; i.e., 5.3%), and vascular ring (*n* = 1; i.e., 2.6%).

**Table 3:** Coexisting congenital defects.

Characteristics	Number	Surgical treatment in infancy
Congenital heart disease (cardiovascular)		
ASD	10	1 (PA banding)
VSD	6	—
AVSD	1	—
PA stenosis	3	—
CoA	2	1
Right aortic arch, vascular ring	2	1
Musculoskeletal		
Vertebral anomalies (butterfly vertebra, vertebral fusion, hemivertebra, additional vertebra, additional ribs)	6	—
Limb deformation (poikilodactylia, tibial hypoplasia)	2	1
Genitourinary		
Polycystic kidney	3	—
Renal agenesis	2	—
Hydronephrosis	1	—
Urachal cyst	1	1
Genital/urinary abnormality	1	1
Gastrointestinal		
Anal atresia/ARM	4	4
Duodenal atresia/annular pancreas	1	1
Cloacal malformation	1	1 (colostomy/vesicostomy)
Pyloric stenosis	1	1
Otolaryngological		
Ears anomalies	4	2
CHARGE syndrome	1	—
Cleft palate	2	2
Laryngeal cleft	1	—
Intracranial/cranial facial		
Corpus callosum agenesis	1	—
Occipital meningocele	1	1
Chromosomal		
Trisomy 21	3	—
Trisomy 18 (Edwards)	1	—

ASD, atrial septal defect; VSD, ventricular septal defect; AVSD, atrioventricular septal defect; PA, pulmonary artery; CoA, aortic coarctation.

Vertebral anomalies (butterfly vertebra, vertebral fusion, hemivertebra, additional vertebra, additional ribs) were present in 6 (15.8%) patients. Limb deformation (poikilodactylia, tibial hypoplasia) was present in two (5.3%) patients.

Congenital defects of the urinary tract were present in six patients: micropenis, renal agenesis, bilateral pelvic kidney, polycystic kidney, hydronephrosis, and urachal cyst (*n* = 1 in each defect).

Gastrointestinal (GI) tract defects were present in five (13.2%) patients, including duodenal atresia/annular pancreas (*n* = 1; i.e., 2.6%), anal atresia/anorectal malformation (ARM) (*n* = 4; 10.5%), cloacal malformation (*n* = 1; i.e., 2.6%), and pyloric stenosis (*n* = 1; i.e., 2.6%).

Ears anomalies were present in four (10.5%) patients, cleft palate in two (5.3%) patients, laryngeal cleft (*n* = 1), and severe tracheomalacia (*n* = 1).

EA with at least two of the following: vertebral defects, anorectal malformation, cardiovascular anomalies, renal malformation or anomalies of upper limbs, in the context of VATER/VACTERL (vertebral, anorectal, cardiac, tracheoesophageal, renal and limb anomalies) association (OMIM: 192350): 9 (23.7%). CHARGE syndrome (OMIM: 214800) (coloboma of the eye, heart defects, atresia of the nasal choanae, retardation of growth and/or development, genital and/or urinary abnormalities, ear abnormalities and deafness) was diagnosed in one (2.6%) patient. Occipital meningocele was present in one (2.6%), and agenesis of the corpus callosum was found in one (2.6%) patient.

### Classification and preoperative prognostic scores

Preoperatively, patients were scored under each of the three classification systems (Waterston, Montreal, and Spitz) (data not shown). After surgical treatment, anatomic findings of EA in patients were categorized using the Gross classification (Table 4).

**Table 4:** Distribution of anatomic findings using the Gross classification categories.

Classification	Groups	Number of patients
Pure esophageal atresia without TEF (type A)	A	3 (7.9%)
Esophageal atresia with proximal TEF (type B)	B	1 (2.6%)
Esophageal atresia with distal TEF (type C)	C	31 (81.6%)
Esophageal atresia with proximal and distal TEF (type D)	D	3 (7.9%)
TEF without esophageal atresia (type E or H-type)	E/H	0

TEF, tracheoesophageal fistula.

With the low rate of mortality (see section: Survival and causes of deaths), all three classification systems (Waterston, Montreal, and Spitz) were found not to be highly valuable at predicting mortality.

### Preoperative management

Preoperative management included patient stabilization and qualification to one-stage vs. two-(multi)-stage approach surgical management. After postnatal cardiologic and respiratory stabilization were achieved, diagnostic imaging defining gap length was performed, and preoperative assessment was done to exclude additional malformations, patients were qualified to one of two groups for planned surgical management: primary or one-stage (Group 1; *n* = 30) vs. two-(multi)-stage approach (Group 2; *n* = 8) (Table 5). The latter one was planned for surgical treatment as soon as possible, but according to clinical stability and concomitant diseases; very low birth weight was an additional but not mandatory criterium.

**Table 5:** Clinical characteristics of one-stage vs. multistage surgery patients.

	Group 1 (one-stage surgery)	Group 2 (multistage surgery)
Number of patients	30	8
Median (quartiles) birth weight (g)	2,580 (2,100–3,240)	2,160 (1,120–2,660)
Median (quartiles) day of final anastomosis	2 (2–3)	13 (10–32) after first surgery 25 (10–39) after birth
Death by day 50	1 (3%)	2 (25%)

### Surgical treatment

**Figure 1:**
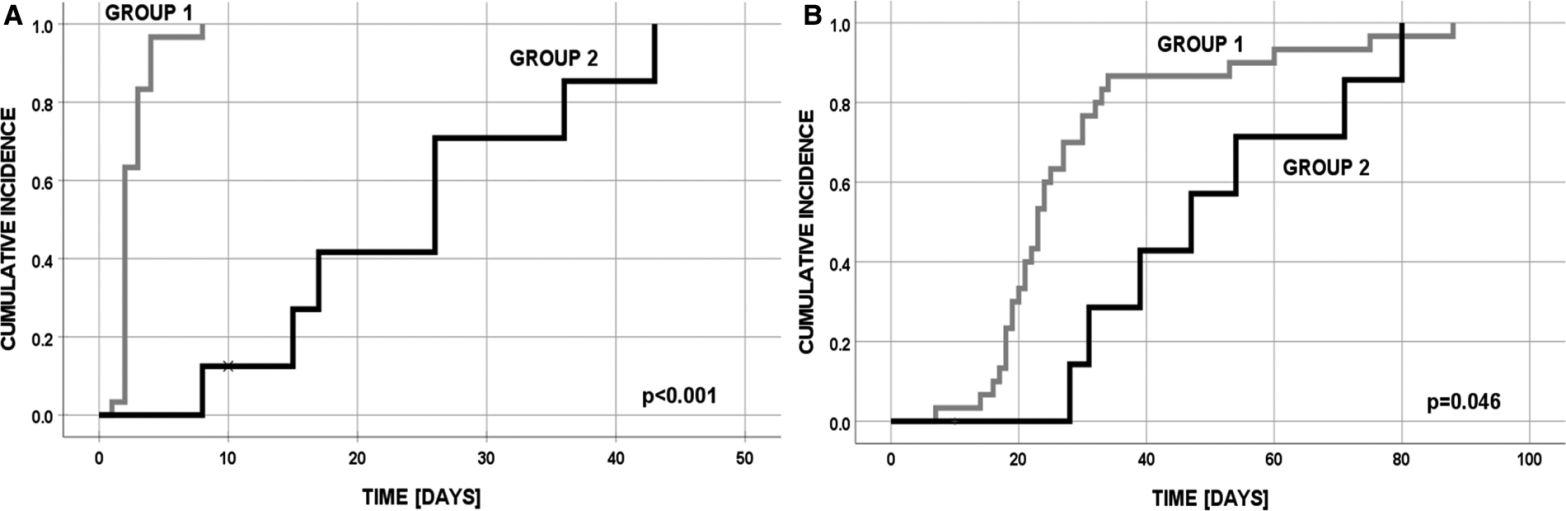
Cumulative incidence in group 1 and 2: (**A**) final EA anastomosis; (**B**) discharge from the hospital (one patient in group 2 has died after first operation at day 10).

Overall, 30 patients underwent primary anastomosis of the esophagus and 8 underwent a multistaged procedure, with or without an initial ligation of the tracheoesophageal fistula and delayed primary anastomosis (Figure 1). None of the 30 patients in group 1 required additional surgical treatment of EA.

In the multistage group 2, the second-stage procedure was performed after the median of 13 (range, 7–42) days. Five patients in overall good condition were operated on within the first month after initial surgery, whereas three patients (3 VLBW infants with birth weight 1,020–1,360 g, week of gestation 28–32), with instable hemodynamic clinical condition and combined congenital heart anomalies (VSD, PDA + ASD, AVSD + CoA + Down syndrome) needed longer time for stabilization (median 32 days interval). Finally, two of these patients have died due to hemodynamic instability, prematurity, pulmonary complications (pneumothorax or massive pneumonia), and liver failure in one case.

In the whole group, a total number of 10 patients underwent gastrostomy creation. Analysis of the multistage group with gastrostomy (*n* = 6) is showing that patients in the “long interval subgroup” needs longer time for stabilization, often having additional general poor status problems to receive full enteral feeding and longer (because of gastrostomy creation) time of initial surgery procedure would additionally negatively affect survival (two deaths in this group). For the “short interval” subgroup the time for esophageal integrity is only approximately 10 days and gastrostomy could (if necessary) be created at the further stages of reconstruction. Patients with gastrostomy from the one-stage group (*n* = 4) presented with severe concomitant neurologic conditions (occipital meningocele, *n* = 1; posthemorrhagic hydrocephalus, *n* = 1; additional patients had problems with oral intake due to severe tracheomalacia, *n* = 1; and bilateral cleft palate, *n* = 1). Two patients from the one-stage group and two patients from the multistage group after dietary and neurologopedic rehabilitation did not further require gastrostomy and had undergone spontaneous or surgical closure of gastrostomy.

Characteristics of parameters of surgical treatment are presented in Table 6. Primary repair and anastomosis of EA/TEF with esophagoesophagostomy and division of the tracheoesophageal fistula was accomplished in 30 infants (78.9%), while eight underwent the staged procedure, with or without an initial ligation of tracheoesophageal fistula and delayed primary anastomosis, including five infants who underwent primary ligation of the fistula, and three underwent staged operative esophageal lengthening (thoracoscopically conducted internal suture traction) with delayed esophageal anastomosis according to the technique described by Patkowski et al. (35, 36).

**Table 6:** Surgery-related factors.

Parameter	Characteristics	Value
Age at first surgery, days	Median (quartiles)	2 (2–3)
One or staged surgery	One-stage	30 (78.9%)
	Two-stage	7 (18.4%)
	Three-stage	1 (2.6%)
Interval between first and second surgery (*n* = 8)	Median (quartiles)	13 (10–32)
Day of final esophageal reconstruction	Median (quartiles)	3 (2–5)
First feeding introduction (day)	Median (quartiles)	8 (7–13)
Extubation (day)	Median (quartiles)	7 (4–15)
Length of ICU stay	Median (quartiles)	9 (6–19)
Length of hospitalization	Median (quartiles)	25 (19–35)
Time of gastrostomy creation (months)	Median (quartiles)	1 (0.8–3.7)
Time of Nissen fundoplication (months)	Median (quartiles)	17 (2–45)
Number of anastomosis calibration (*n* = 34)	Median (quartiles)	1 (1–2)
Additional surgical treatment	Yes	16/38 (42.1%)

ICU, intensive care unit.

Long gap EA is defined as a type A and B esophageal atresia. Of six infants initially managed with ligation of the fistula, four underwent successful delayed primary esophageal repair after staged internal traction procedure, two died prior to undergoing primary repair (one cardiac insufficiency in congenital heart disease and Down syndrome and one multiorgan failure due to extreme prematurity). The remaining two infants underwent dynamic esophageal lengthening with delayed primary esophageal anastomosis as described above (35, 36), of which both achieved primary esophagoesophagostomy after one to two additional operations.

The decision of multistage approach was carried out in eight patients (21%), in case of hemodynamic instability (*n* = 3), severely narrowed, hypoplastic (for 2 cm) distal esophagus (*n* = 1), extremely high localization of proximal esophagus (*n* = 2, including one patient with right-sided aortic arch), and in cases of type A long gap atresia (*n* = 2).

There were no statistical differences between the groups of one-stage vs. multistage EA correction, in the level of proximal esophagus atresia, defined in the preoperative esophageal pouch contrast study (one-stage median 3, quartiles 2–4; vs. multistage median 2.5, quartiles 2–3).

The esophageal anastomosis was conducted under significant tension in 13 (34.2%) patients. Overall, 5–8 stitches (median 6) were used for anastomosis. The postoperative chest tube was used for a median of 4 days (range 2–14). No anastomotic leaks were present in our cohort.

The median time of thoracoscopic procedure in one-stage cases was 150 min (range: 100–305 min), although some of them were associated also with concomitant laparotomy and correction of duodenal atresia (*n* = 1), open gastrostomy (*n* = 2), or colostomy creation (*n* = 1). We noticed generally poor tolerance of anesthesia during surgery in five (13.2%) patients. In 10 patients transient problems in ventilation were noticed, including a mainly transient decrease in saturation (*n* = 6) and one case because of an incidental small tear of the tracheal wall, immediately closed with two simple interrupted sutures. Finally, none of our patients required esophageal replacement.

### Additional surgery during neonatal period

Overall, in 16/38 (42.1%) patients following additional surgical treatments were done: second-stage operation of EA (*n* = 8), cholecystectomy (*n* = 1), or colostomy (*n* = 2). Gastrostomy was performed in 10 (26.3%) patients, at median age of 1 month (range: 3 days—48 months). In 7/10 patients, gastrostomy was closed after correction of other abnormalities (e.g., cleft palate) and dietary rehabilitation. In the whole group, six patients underwent Nissen fundoplication, with three patients aging less than 3 months. Indications for these procedures were as follows: in two patients with type A EA after internal lengthening procedure (2 months and 3 years, respectively) with significant GERD symptoms; in three patients as a part of multidisciplinary treatment of tracheomalacia after readmission because of episodes of apparent life-threatening events (at 1.5, 3, and 17 months, respectively) with significant improvement in two patients and the last patient at age of 5 years with neurological impairment after failed conservative management of GERD with repetitive episodes of aspiration pneumonia.

### Postoperative management

Overall, catecholamines were administered in 23 (60.5%) patients, packed red blood cell transfusion in 13 (34.2%), albumin transfusion in 7 (18.4%), fresh frozen plasma (FFP) in 11 (28.9%), and anti-thrombin III in 5 (13.2%) patients. Postoperative circulatory insufficiency was reported in 10 (26.3%) patients. In one case, circulatory insufficiency was associated with acute kidney injury and hydrothorax.

Regarding nutrition after the operation to establish esophageal continuity: all 38 (100%) infants received total parental nutrition perioperatively, *n* = 36 (95%) received gastric feeding *via* a transanastomotic tube. Gastrostomy tubes were placed in 10 patients (26%), more commonly in patients with Gross type A, B, and D and coexisting defects as compared to type C.

### Complications

#### Complications

Postoperative morbidity occurred virtually in case of each patient (Table 7). The most common complication was recurrent esophageal anastomotic stricture reported in 15/38 (39%) although the median number of dilatations in this group was one calibration. Based on our observations we decided to perform one control dilatation after 4 weeks postoperatively. Infants often needed the help of swallow from a reflex therapist; we found that it was impossible to differentiate if usually, visible stricture is clinically relevant, so with one routine dilatation, clinically relevant stricture was excluded. The need for subsequent dilatation was reported as a stricture. No patient developed neither recurrent fistula nor disruption of the esophageal anastomosis. Surgical site infections were rare. Delayed wound healing without any further consequences was observed after drain removal only in four cases: two cases from the multistage group, and two cases in the one-stage group. In the latter case, both patients were confirmed with septic events.

**Table 7:** Postoperative outcomes.

Outcomes	Value
Number of patients	38 (100%)
Recurrent anastomosis stricture	15 (39.5%)
Fundoplication rate	6 (15.8%)
Gastrostomy	10 (26.3%)
Anastomotic leak	0
Recurrent fistula	0
Mortality	3 (7.9%)^a^
Thoracoscopic method	38 (100%)
Conversion rate	0
Intubation; intensive care unit stay	38 (100%)
Overall postoperative morbidity rate	38 (100%)

^a^ One additional death occurred in different hospital from different cause, 6 months later.

#### Tracheomalacia

Tracheomalacia was assessed as severe only in two cases. In one case despite conducting posterior tracheopexy patient was transferred to the laryngologic center. Moderate tracheomalacia was present in 10 (26.3%) patients, assessed in the postoperative period, after obtaining proper respiratory function.

#### GERD

Gastroesophageal reflux (GERD) was present in 26 patients postoperatively. In 21/26, conservative management was successful. In xi patients the course was severe enough to perform anti-reflux procedure with Nissen fundoplication, which was performed at the median age of 17 (quartiles 2–45) months.

#### Infectious complications

Bacteremia was detected in 10 (26.3%) patients, at median time of 11 days (quartiles, 9–24) after surgery. Etiology included *Staphylococcus epidermidis* (*n* = 5), *Staphylococcus haemolyticus* (*n* = 4), and *Candida albicans* (*n* = 2). In one case mixed infection with *S. haemolyticus* and *C. albicans* was found. All cases of bacteremia occurred among the first consecutive 18 patients. After vancomycin was introduced in prophylaxis, no bacteremia was found for the other 20 (52.6%) patients. Implementation of vancomycin prophylaxis was highly successful in preventing postoperative infectious complications (*p* = 0.002, Fisher’s exact test). Other infectious complications present in the postoperative period included pneumonia (*n* = 3; no pathogen detected), pleuritis (*n* = 2; *Candida albicans, Stenotrophomonas maltophilia*), urinary tract infection (*n* = 1; *Escherichia coli*), conjunctivitis (*n* = 1; *Staphylococcus epidermidis*). Patient with pleuritis developed respiratory distress syndrome associated with pneumonia and bilateral effusions, positive for the presence of *Candida albicans*. In autopsy, the esophageal integrity was confirmed and the anastomosis was healed, on postoperative day +9.

### Survival and causes of deaths

The mortality within 100 days of their esophageal operation was 3/38 (7.9%) including two cases, which were associated with the presence of significant prematurity and congenital heart defects requiring support acute circulatory and respiratory failure. Three deaths occurred on 7, 10, and 47 days after surgical treatment and were caused by multiorgan failure with pneumonia (*n* = 2; one with Down syndrome; one with prematurity) and MRSA (methicillin-resistant *Staphylococcus aureus*) sepsis with thrombosis of vena cava superior. In both patients with pulmonary involvement, complications occurred immediately after transferring to the neonatal intensive care unit (NICU). In both cases, esophageal integrity without recanalization of the fistula was confirmed during the autopsy. Additionally, one patient died at the age of 6 months in another center, due to sepsis (Table 8). With the median follow-up of 4.5 years (quartiles: 1.5–6.4 years), 34/38 patients are alive, and in overall good condition.

**Table 8:** Causes of deaths.

Patient	4	12	26	27
Birth weight (g)	2,600	1,020	1,360	2,550
Additional risk factors	CVC-related sepsis	PDA; extreme prematurity; IVH III grade	Suspicion of cardinal veins pathology; congenital pneumonia	Low birth weight; mother: diabetes mellitus and hypothyroidism
Waterston classification	A	C	C	A
Spitz classification	I	II	III	I
Ladd/Gross classification	C	C	B	C
Post-surgery respiratory insufficiency	0	1	1	0
Gastrostomy	0	1	1	1
Complications		Elevated liver function tests in postoperative period, pulmonary atelectasias	Tension pneumothorax	Severe GERD and tracheomalacia
Cause of death	CVC-related MRSA sepsis, massive SVC thrombosis	Multiorgan failure, prematurity, pneumonia, hemodynamic instability	Multiorgan failure, Down syndrome, cardiac anomaly: Common atrioventricular canal, congenital severe pneumonia, hemodynamic instability	Death related to CVC sepsis in other center
Survival (days)	7	47	10	181

CVC, central venous catheter; PDA, patent ductus arteriosus; IVH, intraventricular hemorrhage; GERD, gastroesophageal reflux disease; MRSA, methicillin-resistant Staphylococcus aureus; SVC, superior vena cava.

### Follow-up

Follow-up was available for 34 patients, with the median observation of 4.5 (quartiles: 1.5–6.4 years). In 27 patients follow-up exceeded at least 2 years. Lansky performance score was determined in children aged over 12 months (Table 9).

**Table 9:** Mid-term follow-up characteristics.

Factor	Characteristics	Value
Lansky performance score (*n* = 32)	Median (quartiles)	90 (80–100)
	Lansky performance score <80	5/32 (15.6%)
	Lansky performance score <90	13/32 (40.6%)
Developmental delay (*n* = 34)	Transient need of rehabilitation support	5/34 (14.7%)
	Cerebral palsy	2/34 (5.9%)
Gastroenterological problems	Dyskinesis (esophageal dysmotility)	13/34 (34.2%)

Overall, in eight patients, developmental delay is observed, including cerebral palsy (*n* = 2) and Down syndrome (*n* = 1), and transient mild impairment (*n* = 5). Episodes of food getting stuck as a sign of esophageal dysphagia were observed in 13/34 (34.2%) at a median of 18 (range: 0–72) months of life. However, 3/13 of those patients have severe abnormalities in neurologic status. In each case without determination as stenosis, full access to the exam and gastroenterologist note after removal of the impacted food was available, and no difficulties with passing the scope with a diameter of 5–7 mm were found. Each patient received an upper GI contrast study after such an event.

## Risk factor analyses

### Length of hospitalization

Hospitalization was longer in the case of the following risk factors: positive blood culture (*p* = 0.036), postoperative respiratory insufficiency (*p* = 0.005), postoperative circulatory insufficiency (*p* = 0.024), packed red cell concentrate transfusions (*p* = 0.008), congenital heart defect (*p* = 0.005), grade C of Waterston classification (*p* = 0.022), and grade II/III of Spitz classification (*p* = 0.005). Patients who received prophylaxis with vancomycin had shorter hospitalization time (*p* = 0.008).

Length of hospitalization correlated with the number of days of intubation (*p* = 0.004, rho = 0.91) and day of final esophageal reconstruction (*p* = 0.004, rho = 0.92); while it was inversely correlated with gestation age (*p* < 0.001, rho = −0.98) and birth weight (*p* = 0.007, rho = −0.89).

### Lansky performance status

Only children over 12 months of age (*n* = 32) were analyzed for performance status. The following risk factors were analyzed for their contribution to performance status, expressed by LPS: neurological impairment, postoperative circulatory insufficiency, preoperative respiratory insufficiency, congenital defects (GI tract, cardiovascular, presence of at least two defects), and length of hospitalization. In univariate analysis, three factors were significant for Lansky Performance Score <80: neurological impairment (*p* = 0.019), length of hospitalization >25 days (*p* = 0.016), and postoperative circulatory insufficiency (*p* = 0.043). In multivariate logistic regression analysis, only neurological impairment significantly contributed to lowering LPS: *p* = 0.009; OR = 36; 95% CI = 2.5 to >100.

### Risk factors for neurological impairment

In univariate analysis, three factors were significant for the risk of neurological impairment: preoperative respiratory insufficiency (*p* = 0.025), congenital cardiovascular defects (*p* = 0.042), and length of hospitalization >25 days (*p* = 0.006). None of them appeared to be significant in multivariate logistic regression analysis, with preoperative respiratory insufficiency showing a weak trend toward significance (*p* = 0.09; OR = 1.5; 95% CI = 0.8–6.3). Length of hospitalization >25 days was a risk factor for gastrointestinal dyskinesis (*p* = 0.044; OR = 1.9; 95% CI = 1.0–5.8).

### Risk factors for overall survival

**Figure 2:**
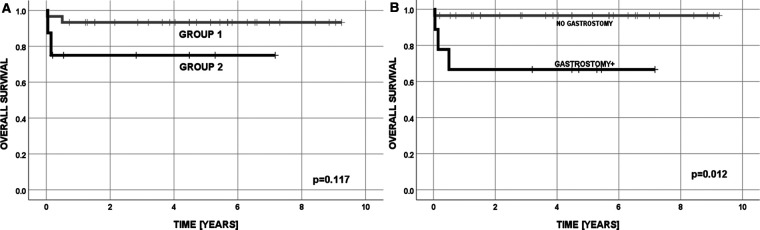
Overall survival: (**A**) in Group 1 and 2; (**B**) with respect to gastrostomy.

Overall survival for all patients was 0.894 ± 0.050, with a mean survival of 8.3 years (95%CI = 7.4–9.2), as determined by the Kaplan–Meier method, and median follow-up 4.5 years. Survival in group 1 was OS = 0.933 ± 0.046 and in group 2 was 0.750 ± 0.153 (Figure 2A).

In univariate analysis (Table 10), two factors were significant for the risk of death: gastrostomy (*p* = 0.012) and Nissen fundoplication (*p* = 0.024). The only factor significant in multivariate logistic regression analysis appeared to be gastrostomy (*p* = 0.033 OR = 14; 95% CI = 1.2–>100), confirmed also by overall survival curves (OS = 0.966 ± 0.034 vs. 0.667 ± 0.157) (Figure 2B).

**Table 10:** Univariate logistic regression of risk factors of overall survival.

Parameter	*p*-value
One-stage surgery	0.138
Bacteremia	0.950
Prophylaxis with vancomycin	0.248
Gastrostomy	0.012
Birth weight <2,800 g	0.470
Symptoms of GERD	0.540
Nissen fundoplication	0.024
Dysmotility	0.197
Dyskinesis	0.204
Use of catecholamines	0.537
Red blood cell concentrate transfusion	0.073
Albumin transfusion	0.321
FFP transfusion	0.333
AT III use	0.417
Waterston grading (A vs. B vs. C)	0.601
Modified Waterston grading (A vs. B vs. C)	0.681
Spitz grading (I vs. II vs. III)	0.101

GERD, gastroesophageal reflux disease; FFP, fresh frozen plasma; AT, anti-thrombin.

## Discussion

In this analysis, we have shown that precise thoracoscopic repair of EA can lead to a high rate of success, not only low mortality but also a low rate of postoperative complications, expressed by no anastomotic leaks or conversions to open technique. Additionally, mortality was not dependent on surgical treatment and was related mainly to coexisting congenital cardiovascular defects, prematurity, very low birth weight, and reasons related to treatment in another hospital due to other causes.

We have shown that thoracoscopic repair of EA resulted in less surgical trauma and a better final effect. We selected a primary repair strategy, which can be performed in most patients, and multistaged repair which can be restricted to unstable patients. With this approach, surgical outcome after primary repair in very low birth weight neonates with type C of EA/TEF is comparable to the outcome in ≥1,500 g neonates. A careful operative view allowed for precise preparation and anastomosis which results in low anastomotic stricture incidence. In our series, the standard of postoperative care contributed significantly to the excellent postoperative outcome, and the presence of prematurity was a limited disadvantage.

The very important achievement of our study was the absence of anastomotic leakage. A variety of factors have contributed to this effect, namely: delicate manipulation during mobilization and suturing; minimal touch technique; minimal mobilization of the distal esophagus; relatively small amount of stitches (usually 5–7); a wider field of vision in thoracoscopic surgery, which is beneficial for separating the esophagus; and selection of multistage operation approach in case of excessive tension. It seems that the endoscopic approach has the potential to lower the incidence of anastomotic leakage than after open surgery for EA. However, a recent meta-analysis Borruto et al. revealed no statistically significant differences in the complications or outcomes between these two methods (37).

Esophageal leakage is reported as one of the most common and severe complications following EA/TEF surgery with an incidence of approximately 5%–25%. A variety of factors can contribute to the leakage, including excessive mobilization of the distal end of the esophagus, esophageal injury, poor suturing technique, anastomotic tension, the use of inappropriate sutures, associated ischemia, a long gap length or sepsis (6, 19, 20, 22, 23, 38).

Thoracoscopic repair of esophageal atresia has become a new standard and a possibility of a high level of neonatal surgery (39–41). Introducing MINT (A minimally invasive no-touch technique for EA correction) with two-stage anastomosis decision-making readiness results in lower rates of complications in relation to open surgery. Endoscopic treatment of EA provides better vision control and changes surgical technique (35). We have also found that the first-line strategy for the repair of EA without the use of a gastrostomy is also beneficial for overall survival. However, this management in the case of “difficult esophageal atresia” in true long gap esophageal atresia is still a challenge (42). Nevertheless, more data with detailed analysis of postoperative management are needed as anastomotic leakage is a common problem in different hospitals.

Approach with final esophagus anastomosis in two or three stages did not influence additional complications. We can hypothesize that the multistage approach possibly prevented some complications e.g., doubtful unsecure anastomosis, leaks, disbandment, thus this approach could contribute to the improved outcome and future prognosis, regardless of prognostic classifications (e.g., type C). Additionally, there were no patients where further nonanatomic esophageal reconstruction procedures like gastric pull-up or intestinal reconstruction were necessary.

Our experience indicates that in cases of prolonged time of surgery of difficult EA anatomy and significant tension of both parts of the esophagus, it is safer to go to a staged procedure, after securing the esophageal traction stitches and final stabilization of the patient clinical condition.

Our results in this group of patients revealed, that in case of “difficult anastomosis,” taking into consideration the priority of the patient’s safety, it is better to perform the staged procedure of esophageal anastomosis. In our opinion, a minimally invasive thoracoscopic approach offers good local conditions during the second stage of the procedure with minimal after-effects for the patients. These results of the one-stage vs. two-stage approach indicate a high patient safety profile and good tolerance of second anesthesia and effective surgical outcome.

Nevertheless, even in the case of a two-stage approach, a poor outcome may occur. Our patient with VLBW and Down syndrome having pneumonia and heart insufficiency has developed a respiratory distress syndrome associated with pleuritis and bilateral effusions, positive for the presence of *Candida albicans*. In autopsy on postoperative day +9, the esophageal integrity was confirmed and the anastomosis was healed. In this case, colonization and neonatal fungal sepsis overlapped with congenital abnormalities and finally contributed to poor outcomes (43–46), while esophageal anastomosis was secure and intact.

Another important finding of our study is the value of introducing prophylaxis with vancomycin, which resulted in a significantly lower rate of postoperative infections and contributed to a shorter period of ICU stay and time of hospitalization. This observation even if not novel, provides a rationale for selecting an antibiotic strategy in the management of selected newborns with congenital anomalies (31, 32).

We also presented results of long-term outcomes. With a median follow-up of 4.5 years, almost 85% of patients have a Lansky performance score ≥80, thus being able for normal activity. Another important long-term value is quality of life (QoL), not analyzed in our study. Rozensztrauch et al. have shown that the QoL of children with EA born before the 37th week of gestational age was lower in social functioning than children born in term; however, the presence of concomitant anomalies contributed to decreased QoL (47).

The limitation of our study is the number of patients. However, due to excellent results in terms of no anastomotic leakage and no necessity conversion to open surgery, both in one-stage and multistage approaches, we decided to analyze the outcomes of endoscopic treatment of EA in a single-center study. We experienced the effect of the “learning curve” in the area of longer surgery time at the start of the program but it did not influence the final results. Another factor that influenced the lack of a learning curve depending on complications was the fact that the first four patients had birth weights of more than 2,500 g, being at a lower risk of fatal complications. On the other hand, experience with CVC-related infections, leading to the prophylactic use of vancomycin, was evidence of a learning curve in supporting therapy. A number of issues remain to be solved, including optimal management of extremely low birth weight neonates, optimal timing for anastomosis in case of staged repair in an unstable patient, optimal management in pneumothorax, and possible reduction of the operative time.

The mortality rate in our study was low, and no child died due to surgical reasons. Continuous improvement in surgical techniques leads to a decrease in mortality from EA (6, 19, 20, 22, 23). In this context, the value of existing mortality classification systems, although very practical and informative, is lower nowadays in predicting outcomes (3, 48, 49). Nevertheless, classification systems are useful for comparing outcomes and prenatal consultations to explain to parents the likelihood of their child surviving and what factors can influence the outcome. Currently associated cardiac and chromosomal anomalies and preoperative ventilator dependence and severe associated anomalies could be more important for survival than body weight (50–53).

In conclusion, we have found that the outcome of patients with EA treated with the thoracoscopic method is better than with previously reported approach in terms of surgery-dependent morbidity in the context of a lower rate of anastomosis leakage and other complications, such as conversion to open surgery, regardless of the prognostic criteria of the classification system.

## Data Availability

The original contributions presented in the study are included in the article/Supplementary Material, further inquiries can be directed to the corresponding author.
